# Nonlinear EEG parameters of emotional perception in patients with moderate traumatic brain injury, coma, stroke and schizophrenia

**DOI:** 10.3934/Neuroscience.2018.4.221

**Published:** 2018-11-07

**Authors:** Galina V. Portnova, Michael S. Atanov

**Affiliations:** 1 Institute of Higher Nervous Activity and Neurophysiology of RAS, 5A Butlerova St., Moscow 117485, Russia; 2 The Pushkin State Russian Language Institute

**Keywords:** EEG, fractal dimension, EEG envelope, Hjorth, schizophrenia, stroke, coma, traumatic brain injury

## Abstract

**Objective:**

The aim of this study was to determine the EEG changes induced by emotional non-verbal sounds using nonlinear signals' features and also to examine the subjective emotional response in patients with different neurological and psychiatric disorders.

**Methods:**

141 subjects participated in our study: patients after moderate TBI, patients in acute coma, patients after stroke, patients with schizophrenia and controls. 7 types of emotionally charged stimuli were presented. Non-comatose participants were asked to assess the levels of experienced emotions. We analyzed fractal dimension, signal's envelope parameters and Hjorth mobility and complexity.

**Results:**

The Hjorth parameters were negatively correlated with irritation. The fractal dimension was positively correlated with arousal and empathy levels. The only presentation of laughter to post-stroke patients induced the reaction similar to the control group.

**Conclusions:**

The results showed that the investigated nonlinear features of resting state EEG are quite group-specific and also specific to the emotional state.

**Significance:**

The investigated features could serve to diagnose emotional impairments.

## 1. Introduction

In this study we examined a set of time-domain and nonlinear features of EEG signal in patients with different neurological and psychiatric disorders. These features were shown to differ in patients with neurological disorders such as epilepsy, attention-deficit/hyperactivity disorder (ADHD) and Alzheimer's disease [1]. So, we expect that the complexity and chaotic nature of EEG data are useful to discriminate these disorders by their specific emotional traits. As reported by some researchers [2], quantitative measures of chaos and nonlinear features characterize electrophysiological abnormalities in neuropsychiatric disorders that are not evident in linear analysis. To show the effectiveness of these features to diagnose a concussion, in an approach similar to power analysis, the features can be calculated for both usual EEG frequency bands (rhythms) and individual EEG frequencies (2-Hz wide bands). This method came from a study on concussed athletes [2]. The investigated signal features: time domain Hjorth parameters, approximate entropy, and the Hurst exponent, didn't differ between the groups of the athletes and their healthy peers if calculated in the rhythm-band filtered case but they did differ in the narrow-band filtered case.

The observation of significantly different nonlinear features also revealed important notions about concussed athletes: they exhibited a decrease in Hjorth complexity and mobility. It has been reported by Pezard with colleagues [3] that depressive subjects tend to display lower complexity than controls. Moreover, it was reported that decreased complexity and mobility are associated with insomniac subjects [4]. Approximate entropy quantifies the amount of regularity in the data by calculating the upcoming amplitude values of the signal based on the knowledge of the preceding amplitude values [5]. Sohn with colleagues [6] reported about significantly lower approximate entropy for a group of ADHD subjects compared to matched controls and hypothesized that the patients might not have sufficient levels of cortical activation to reach the requirements for attention-demanding tasks. Following their hypothesis, the approximate entropy is discriminative in this case, we suppose it reflects the altered cortical information processing. Moreover, pathological disorder studies on schizophrenia, posttraumatic stress disorder, panic disorder, and epilepsy have reported lower Hjorth complexity in pathological states compared to healthy subjects [7]. We claim that lower EEG complexity is attributed to the abnormal neural integration in the above-mentioned mental disorders.

Nonlinear EEG parameters were previously shown to be related to emotional states and affective reactions. For example, fractal dimension of EEG signals provides comparative performance for both facial [8] and auditory [9] emotion recognition. Differential entropy of EEG signals proved to be more suitable for emotion recognition than traditional frequency domain EEG features [10]. Hjorth parameters can be used for happiness/sadness recognition of visual stimuli [11] and emotional components of music [12]. Thus, nonlinear parameters are a good choice in the paradigm of emotional processes investigation.

Nonlinear features are also a good tool for emotional processes research in neurological and psychiatric disorders of our interest. For example, fractal dimension decreases in the acute phase of a stroke and was also associated with worse clinical recovery [13]. Other researchers have reported that spectral entropy reflects the slowing of brain activity in patients with post-stroke vascular dementia and stroke-related cognitive impairment, whereas permutation entropy and Tsallis entropy reflect the complexity of the examined signals [14]. Using the support vector machine algorithm Chu and co-authors [15] even succeeded in classifying the type of induced emotion by EEG signal in groups of patients with moderate and severe schizophrenia and their healthy counterparts.

In our studies, we attempt to determine the specificity of emotional auditory nonverbal perception using nonlinear EEG parameters. In the present study we investigated four groups of patients with different diagnoses and therefore different types of emotional impairment, i.e., patients after: severe TBI (in coma), moderate TBI, stroke, and with schizophrenia. It is well known that mood depression is a typical post-stroke complication [16] and is associated with a disability [17] and with a worse rehabilitation outcome in stroke survivors [18]. Patients with moderate TBI demonstrate significant emotional and behavioral maladjustment, increased difficulties with anger management, antisocial behavior and abnormal self-monitoring [19]. Severe TBI is often accompanied by the lack of empathy, reduced behavioral regulation and impaired social function [20]. In our previous research on patients with TBI we found the EEG response on emotional stimulation to vary depending on the severity of trauma [21] Namely, patients with severe TBI showed decreased theta-rhythm power in the frontal areas during unpleasant sounds presentation, while the patients with moderate TBI exhibited increased alpha-rhythm power in the occipital areas during both pleasant and unpleasant stimulation. Patients with schizophrenia usually lack outward expression of emotion, diminished motivation and reduced experience of pleasure [22]. The described emotional changes in different groups of patients are polymorphic and have different origins and pathogeneses. We expect that nonlinear features of EEG signals could reveal the dynamics accompanying the emotional processes and discriminate these groups of patients.

## 2. Materials and methods

### 2.1. Participants

5 groups of adult subjects participated in our study (N = 141): healthy (control group), patients after moderate TBI (mTBI), patients in acute coma (caused by severe TBI), patients after stroke in the left middle cerebral artery and patients with schizophrenia. The exclusion criteria were: any history of epileptic seizures, tumors, and massive brain hematomas, additional medications or accompanying diseases. The resulting structure of the groups is depicted in Table 1. Glasgow Coma Scale was used for diagnostic purposes only.

Access to patients after TBI by the Central Clinical Hospital of Russian Academy of Science, access to patients with schizophrenia was provided by the 1st Psychiatric Clinical Hospital named by N.A. Alexeev.

No participants took any anticonvulsants, nootropics or antianxiolytics. All the comatose patients received identical acute care therapy according to local standards (the medication's doses were similar for each patient). All participants with schizophrenia received medical therapy of haloperidol (the same medication's doses). Patients after stroke received antihypertensive therapy (the medication's doses were similar for each patient). Patients after mTBI and healthy subjects were not under medication.

The outcome of comatose patients was as follows: 6 patients recovered within two weeks of the study, 8 patients remained in a vegetative state for the following three months, 5 patients were transferred to other hospital departments due to extracranial complications.

We analyzed hearing function in healthy subjects and patients after moderate TBI as we presented auditory stimulation. We used PDD-401 audiometer (Piston Ltd., Budapest, Hungary) to identify hearing threshold levels. None of the examined subjects had any symptoms of hearing loss.

**Table 1.** Descriptive statistics of subjects.

Groups	Number of subjects	Age	Sex (f/m)	Glasgow Coma Scale	Duration of the disease
Control group	37	31.6 ± 7.1	22/25	-	-
Patients with schizophrenia	26	25.9 ± 4.7	11/15	-	3 ± 0.6 years
Moderate TBI patients	31	31.2 ± 6.6	15/16	13.0 ± 1.9	10.5 ± 7.7 months
Patients after stroke	28	55.2 ± 5.1	13/15	-	7.9 ± 1.3 months
Patients in coma	19	27.1 ± 5.3	8/11	4.3 ± 1.7	5.9 ± 2.9 days

Note: Mean ± std.dev. where applicable.

All participants (or their authorized relatives) gave written informed consent prior to participating in the study. The protocol of the study was approved by the Ethics Committee of the Institute of Higher Nervous Activity and Neurophysiology of RAS.

### 2.2. Stimuli

We presented 30 second long sounds as stimuli. There was initially a large list (about 40) of such sounds, then a group of 198 healthy experts (mean age about 30 years) assessed all the stimuli on scales of pleasantness, arousal, fear, gladness, etc. We selected 7 stimuli for this study—most emotionally charged with most stable assessment: a dog barking, a crying infant, vomiting, coughing, scratching nails on a glass, a bird singing, and human laughter. Each stimulus was presented randomly 8 times, the interstimulus interval was 700–2000 msec long (randomly conducted): the obtained ∼ 240 sec EEG fragments for each stimulus were further analyzed. Resting states with open and closed eyes were recorded for 2 minutes in the beginning and at the end of the study.

The subjects of the control, schizophrenia, stroke and mTBI groups assessed emotional valence and arousal level of the stimuli by the scales pleasantness (from −5 to 5), arousal (from 0 to 10), fear (from 0 to 10), empathy (from 0 to 10) and irritation (from 0 to 10). The stimuli were presented in a random order for 30 seconds (8–10 times each) with 0.7–2.0 second gaps between them (Table S1); the whole experiment lasted about 50 minutes.

### 2.3. EEG registration

The subjects sat in a comfortable position in an armchair in an acoustically and electrically shielded chamber, the comatose patients laid in a hospital bed in a resuscitation unit. The participants were instructed to remain calm and to listen to the presented sounds keeping their eyes closed (to avoid visual interference) and not to fall asleep. The stimuli were presented via earphones. EEG was recorded using the “Encephalan” (Medicom MTD, Taganrog, Russian Federation) device. Polygraphic channels were also recorded although these data are not presented. 19 AgCl electrodes (Fp1, Fp2, F7, F3, Fz, F4, F8, T3, C3, Cz, C4, T4, T5, P3, Pz, P4, T6, O1, O2) were placed according to the International 10–20 system. The electrodes placed on the left and right mastoids served as joint references under unipolar montage. The vertical electrooculogram (EOG) was measured with AgCl cup electrodes placed 1 cm above and below the left eye, and the horizontal EOG was measured with electrodes placed 1 cm lateral from the outer canthi of both eyes. The sampling rate was 250 Hz, the filtering was set to bandpass 1.6–30 Hz. The electrode impedances were maintained at less than 10 kΩ. The small artifacts were deleted manually. EMG-related artifacts were deleted manually and using filtering 1.6–30 Hz. Eyes movement artifacts were cleaned out using EOG data by Encephalan device.

## 3. Data analysis

The artifact correction was conducted by the following way: the small artifacts were deleted manually, eyes movement artifacts were cleaned out using EOG data by “Encephalan” device.

### 3.1. Fractal dimension (FD)

We conducted the calculations of the examined signal bandpass-filtered in the range of interest (2–20 Hz), Butterworth filter of order 12 was used. FD was evaluated using the Higuchi algorithm [23].

### 3.2. Envelope mean frequency (EMF)

To express the (de-)synchronization dynamics of the rhythms we applied the following method. First, we calculated the envelope of the EEG signal for the whole frequency range (1.6–30 Hz) and for the alpha rhythm (8–13 Hz) using the Hilbert transform [24]. Second, we assessed the (in-)stability of the envelope's amplitude by calculating its average frequency using FFT (wideband—EMF, alpha—EMFA) and the ratio of its standard deviation to its mean (wideband only—RAT).

### 3.3. Hjorth parameters

Hjorth complexity [25] represents the change in frequency and indicates how the shape of a signal is similar to a pure sine wave. This parameter was calculated for wideband 1.6–30 Hz filtered signal in the following way: 
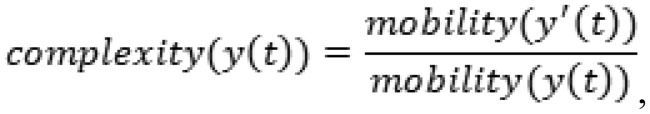
 where 
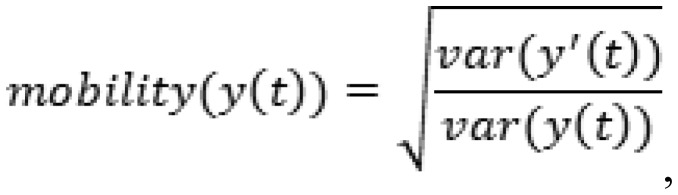
 y(t)—a signal, y'(t)—its derivative, and var(...)—the variance.

The Hjorth complexity and mobility showed very similar results in our research, so we refer to them both as Hjorth parameters.

### 3.4. Power spectral density

Fast Fourier Transform (FFT) was used to evaluate the PSD, the resulting data were integrated over intervals of unit width in the range of interest (2–3 Hz, 3–4 Hz, …, 19–20 Hz). It was carried out for each EEG fragment and then used in correlation analysis with nonlinear EEG features.

### 3.5. Statistical analysis

A repeated-measures ANOVA with Bonferroni correction for multiple comparisons (*p* < 0.05) was used to determine group effects on EEG metrics (post-hoc Tukey). We analyzed separately group effect for non-linear features for the resting state and the group effect of differences between the features during stimulation was compared to the values during rest. The Pearson correlation coefficient between PSD and nonlinear features was calculated. Only significant (*p* < 0.05) correlation values were used for further analysis.

## 4. Results

### 4.1. Power spectral density

**Figure 1.**
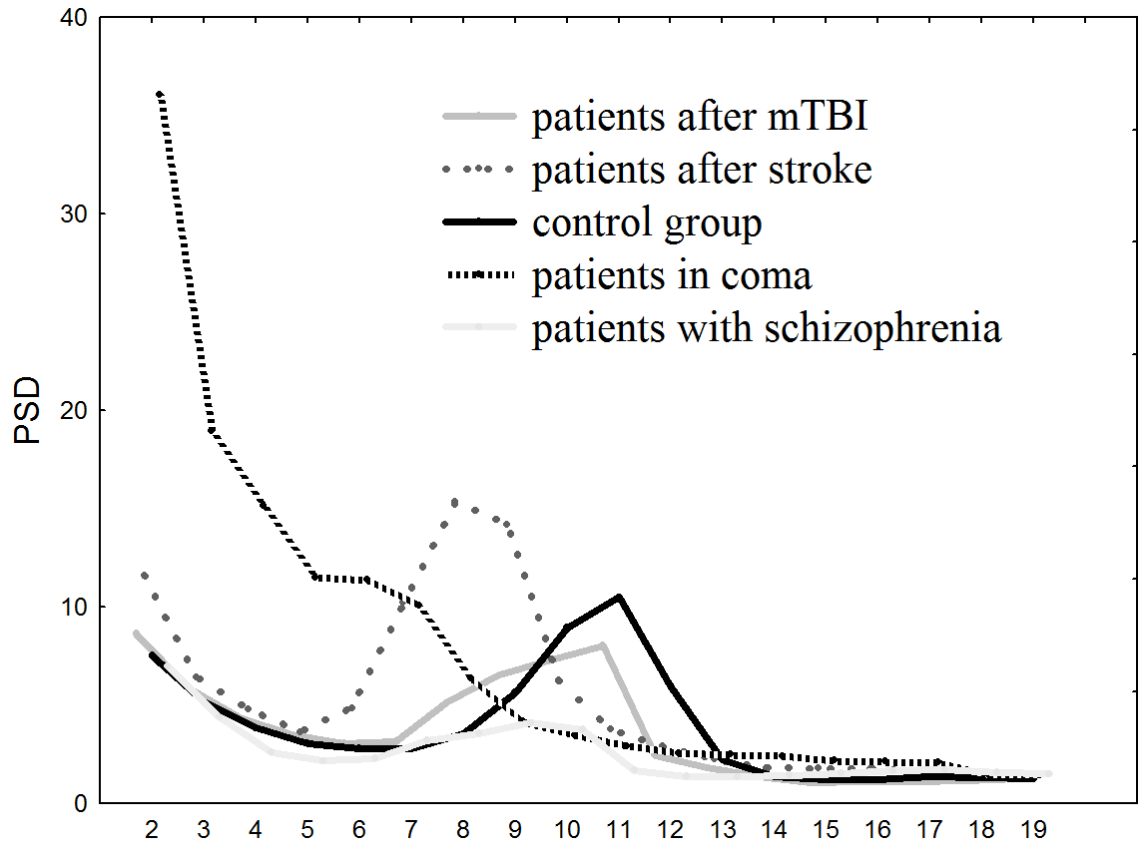
PSD values 2–20 Hz averaged over electrodes C3, Cz, C4: x—frequency in Hz, y—PSD in µV^2^/Hz.

Comatose patients had higher 2–7 Hz PSD compared to other groups of subjects (F(4, 141) = 8.928, *p* < 0.00001) in all electrodes. Patients after stroke had higher 5–9 Hz PSD compared to TBI, schizophrenia and control groups (F(4, 141) = 10.318, *p* < 0.00001) (except Т5, Т6 electrodes). The alpha-rhythm PSD (10–13 Hz) was significantly higher in control group (F(4, 141) = 7.0168, *p* < 0.0001) in central, parietal and occipital areas: electrodes C3, Cz, C4, P3, Pz, P4, O1, O2 (depicted in Figure 1).

### 4.2. Group differences during rest

In this subsection the values during rest were compared between the groups.

#### 4.2.1. FD

1. Lower in patients after stroke (except C3, T4, P3, Pz, P4), F(4, 141) = 3.47050, *p* < 0.03.
2. Higher in patients with schizophrenia (C3, T5, P3, Pz, P4, T6, O1, O2), F(4, 141) = 4.7050, *p* < 0.005.

#### 4.2.2. EMF

1. Lower in patients after stroke (all except F7, C3, P3, Pz, P4, O1).
2. Lower in comatose patients (all except P4, T6).
3. Higher in patients with schizophrenia (all except C4, T4, T6), F(4, 137) = 5.2412, *p* < 0.001.

#### 4.2.3. EMFA

Higher in patients with schizophrenia (Fp1, Fp2, F7, F3, Fz), F(4, 137) = 5.9452, *p* < 0.0005.

#### 4.2.4. RAT

Higher in comatose patients (Fp1, F7, Fz, T3, C3, T5, P3, Pz, O1, O2), F(4, 141) = 5.97050, *p* < 0.001.

### 4.3. Hjorth parameters

Both mobility and complexity were lower in comatose patients (all except F3, F4, T5), F(4, 141) = 6.1986, *p* < 0.001.

Notably, no feature differed between mTBI and control groups.

**Figure 2.**
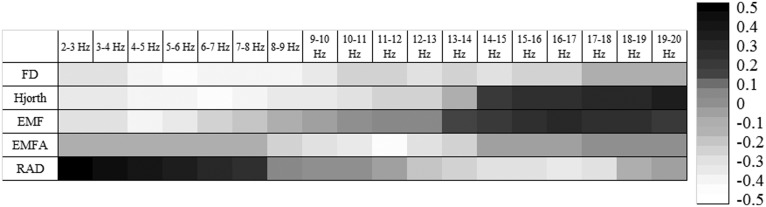
Significant (|r| > 0.35, *p* < 0.05) correlations between PSD and nonlinear EEG features.

The correlation analysis between linear and nonlinear parameters showed that FD was negatively correlated with 2–10 Hz and 13–14 Hz PSD, Hjorth parameters were negatively correlated with 2–8 Hz PSD and positively correlated with 15–20 Hz PSD. EMF was negatively correlated with 2–6 Hz PSD and positively correlated with 15–19 Hz PSD. EMFA was negatively correlated with 10–12 Hz PSD. RAD was positively correlated with 2–8 Hz PSD and 16–17 Hz PSD (depicted in Figure 2).

### 4.4. Group differences during stimulation

The features during stimulation were compared to the values during rest.

#### 4.4.1. FD

1. Higher in control group during presentation of crying (except F7, C3, Cz, C4, O2), *p* < 0.03.
2. Lower in patients with moderate TBI during presentation of coughing and vomiting (Fp1, Fp2, F7, Fz, F3, F4, C3, C4), *p* < 0.009.
3. Higher in comatose patients during these stimuli (Fp1, Fp2, F7, F3, Fz, F8, O1, T4), *p* < 0.03.
4. Higher in patients with schizophrenia during animal sounds presentation (barking and singing) (Fz, Fp2, F8, Cz, F4, C4, T4, P4, T6, O1), *p* < 0.04.
5. Lower in patients after stroke during animal sounds (barking and singing) presentation (F4, Fz, Pz, P4), *p* < 0.01, and higher during presentation of laughter (Fp1, Fz, F3, T3), *p* < 0.02.

#### 4.4.2. EMF

1. Lower in control group during vomiting, coughing and scratching presentation (except T4, P4, T6), *p* < 0.05.
2. Lower in patients with moderate TBI during scratching (P4,T6, Cz, C4), *p* < 0.007.
3. Lower in comatose patients during vomiting, coughing and scratching presentation (Fp2, F4, F8, T4, T6, P4), *p* < 0.03 and higher during laughter and crying presentation (F4, F8, C4, T4, Pz, P4, T6, O1, O2).
4. Higher in patients with schizophrenia during bird song presentation (except Fp1, T3, T5, O2), *p* < 0.009.

#### 4.4.3. EMFA

1. Lower in control group during presentation of vomiting, coughing, scratching, bird singing and barking (all channels).
2. Higher in patients with schizophrenia during presentation of bird singing, laughter, crying, coughing and vomiting (all except O2) and barking (Cz, C4, P4, T6).

#### 4.4.4. RAT

1. Higher in patients after moderate TBI during presentation of crying (Cz, C4, T4,T6), *p* < 0.04 and laughter (C3, Cz, C4, T4), *p* < 0.05.
2. Higher in comatose patients during presentation of pleasant stimuli (laughter and bird singing) (F8, Cz, T6, Pz, O1, O2), *p* < 0.02.
3. Higher in patients with schizophrenia during presentation of coughing, laughter, scratching and bird singing (except T3, C3, T5, T6, O1, O2), *p* < 0.03, and lower during presentation of crying and barking (F3, T3, C3, Cz, C4, Pz, O1, O2), *p* < 0.01.

### 4.5. Hjorth parameters

1. Lower in patients after moderate TBI during all emotional stimuli (all channels), *p* < 0.05.
2. Lower in comatose patients during presentation of bird singing, barking and crying (F7, T3, C3, C4, P4, P3), *p* < 0.01.
3. Lower in patients with schizophrenia during presentation of laughter, barking and crying (Fp1, Fz, F3, F8, Cz, P3, Pz), p < 0.03, and higher during presentation of scratching (P3, Pz, O1), *p* < 0.04.
4. Lower in patients after stroke during presentation of barking (Fz, C3, Cz, Pz), *p* < 0.05.

The results of this subsection are summarized in Table 2.

**Table 2.** Significant differences of the features between stimuli presentation and rest (“+”—higher in rest, “−”—lower in rest).

	Bird song					Barking					Crying				
	FD	EMF	EMFA	RAD	Hjorth	FD	EMF	EMFA	RAD	Hjorth	FD	EMF	EMFA	RAD	Hjorth
Control group			−								+				
Schizophrenia	+	+	+	+		+			−	−			+	−	−
Moderate TBI					−					−				+	−
Stroke	−					−				−					
Coma				−	−					−		+		−	−

	Coughing and Vomiting					Scratching					Laughter				

Control group	+	−	−				−	−							
Schizophrenia			+						+				+	+	−
Moderate TBI	−				−		−			−				+	−
Stroke											+				
Coma	+	−					−					+			

**Figure 3.**
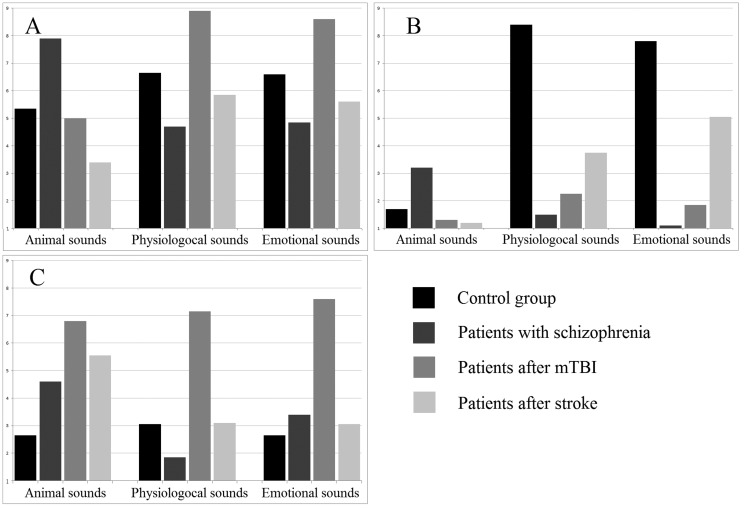
Emotional assessment of stimuli. A—arousal (0 10), B—empathy (0 10), C—irritation (0 10).

The assessment of emotional stimuli showed that participants of the control group reported significantly higher pleasantness of laughter (F(4, 141) = 7.124, *p* < 0.0005) and lower pleasantness of crying (F(4, 141) = 3.945, *p* < 0.05), compared to the groups of patients (depicted in Figure 3). Arousal was significantly higher in mTBI group during the presentation of unpleasant stimuli (coughing, vomiting) compared to other groups of subjects (F(4, 141) = 8.61, *p* < 0.0001).

### 4.6. The correlations between emotional assessment of stimuli and non-linear features

The empathy values (Table S1) were positively correlated with FD in the control group during the presentation of all stimuli (r > 0.46, *p* < 0.05). The arousal values were positively correlated with FD in patients with schizophrenia during the presentation of animal sounds (barking and bird singing) (r > 0.37, *p* < 0.05). The irritation values were negatively correlated with Hjorth parameters for all types of stimuli in all groups (r < −0.39, *p* < 0.05).

## 5. Discussion

In the present study, we found that the examined nonlinear features of resting state EEG are quite group-specific. Particularly, RAT was higher, Hjorth parameters and EMF were lower in comatose patients; EMF and FD were lower in patients after stroke. Having the correlation of these parameters with delta- and theta-rhythm PSD we suppose that the found specificity is related to the higher slow-wave activity [26],[27] in these patients. These results match the previous data about reduced FD in patients with Alzheimer's disease [28] and studies reported that spectral entropy reflects the slowing of brain activity in post-stroke vascular dementia and stroke-related cognitive impairment patients [13].

EMFA and FD were oppositely higher in patients with schizophrenia. The negative correlations of these parameters with alpha-rhythm PSD may support the previous data of unusual alpha-rhythm activity of EEG in this group of patients [29]. This activity also proved to be related to clinical symptoms [30]. EMFA was shown to be sensitive to the emotional reaction of patients with schizophrenia: we observed the increase of EMFA during emotional stimulation only in this group, it even decreased in control group and demonstrated no significant changes in other groups. We supposed that the unusual emotional perception of patients with schizophrenia caused the abnormal changes in the EEG nonlinear parameters. Further, alpha activity during coma is not the same as physiological alpha rhythm [31] and patients after stroke and traumatic brain injury appeared to show a decreased frequency and magnitude of the alpha-rhythm [32], therefore EMFA didn't reflect their EEG changes.

We also found that some nonlinear EEG parameters can be associated with certain emotional states. For example, the control group showed an increase of FD during stimuli presentation (crying, coughing, and vomiting) which evoked arousal and empathy. These FD values also correlated with the subjective assessment of these emotions levels during the presentation. Some previous studies have reported that higher emotional response is associated with higher FD [33]; the “acute” or sudden emotions can also be determined using FD [34]; furthermore, FD was found to be useful to detect the level of arousal [35]. In our previous study [36] we also observed a significant positive correlation between FD of the EEG signal and fMRI BOLD-signal in some regions including the limbic system. Thus, FD in the control group could be used as a measure of arousal. We also observed this “normal” response in patients after stroke during laughter presentation, which was correlated with arousal feelings. The animal sounds (bird singing and dog barking), which evoked mostly irritation feelings in this group of patients, were inversely accompanied by a decrease in FD.

FD increase in patients with schizophrenia was similarly accompanied by higher reported arousal (bird singing and dog barking). The higher FD in these patients has previously been explained by other effects like self-focused emotions [37] and neurotic behavior [38], and the latter was also shown to be correlated with higher FD during auditory stimuli presentation. Thus, the higher FD in patients with schizophrenia should be explained not only by their more intense emotional reaction, i.e. higher arousal, but also by the loosened organization of thoughts and mental suggestions of certain superior abilities [39].

Hjorth parameters showed a significant decrease in all groups of patients during stimulation, but not in healthy subjects. This effect was most pronounced in comatose patients in response to animal sounds (bird singing, dog barking) and crying. Hjorth parameters were previously shown to be useful for recognition of emotional state: happy, sad, neutral and afraid [11], though using pictures as stimuli. We expected we could determine the pleasantness of our stimuli but the changes in the parameters didn't represent pleasantness in any way. Moreover, our results showed a negative correlation of Hjorth parameters during stimulation with the irritation level, which was most demonstrative considering barking sounds.

This study has some limitations which have to be pointed out. First of all, in spite of controlled medication's dozes, we are not sure that the emotional assessment and accompanying EEG changes in patients with schizophrenia were related only with their emotional disturbances. We also assume that the expanded amount of emotional sounds (we presented only seven types) and the presentation of emotional stimulation in other modalities could provide more detailed information about emotional impairment in patients.

## 6. Conclusions

We've shown that FD can be used to detect arousal in response to emotional stimulation.

EMFA reaction to the stimulation was observed only in patients with schizophrenia (increase) and controls (decrease).

The emotional stimuli (coughing and vomiting) that induced empathy in healthy subjects induced not empathy but irritation in patients with moderate TBI and led to opposite FD changes in this group. We've found that the presentation of laughter induced the same EEG changes in patients after stroke and comatose patients as in control group.

Hjorth parameters are useful to detect the abnormal increased irritation during stimulation.

In upcoming research we are planning to build a classification procedure to diagnose emotional impairments in different psychical and neurological diseases using these nonlinear parameters.
