# Supplementary material for: Nonlinear EEG parameters of emotional perception in patients with moderate traumatic brain injury, coma, stroke and schizophrenia
Source: AIMS Neurosci. 2018 Nov 7;5(4):221–35. doi: 10.3934/Neuroscience.2018.4.221 (PMC7179336; doi:10.3934/Neuroscience.2018.4.221)
Supplement: Supplementary file 1 [file neurosci-05-04-221-s001.pdf]

AIMS Neuroscience

http://www.aimspress.com/journal/neuroscience

AIMS Neuroscience, 5 (4): 221–235.
DOI: 10.3934/Neuroscience.2018.4.221
Received date: 05 September 2018
Accepted date: 21 October 2018
Published date: 07 November 2018

Research article

Nonlinear EEG parameters of emotional perception in patients with moderate traumatic brain injury, coma, stroke and schizophrenia

Galina V. Portnova[1,2,*] and Michael S. Atanov [1]

[1] Institute of Higher Nervous Activity and Neurophysiology of RAS, 5A Butlerova St., Moscow 117485, Russia
[2] The Pushkin State Russian Language Institute

* **Corresponding Author:** Email: caviter@list.ru; Tel: +7-495-334-7000; Fax: +7-499-743-0056.

Supplementary

Table S1. Subjective assessment of stimuli.

		Bird singing	Coughing	Vomiting	Crying	Laughter	Barking	Scratching
Control	Pleasantness (−5 5)	4.7 ± 0.2	−4.6 ± 0.9	−4.7 ± 0.7	−4.5 ± 0.3	4.1 ± 0.2	−4.6 ± 0.3	−4.5 ± 0.8
	Arousal (0 10)	2.8 ± 0.5	6.8 ± 0.8	6.5 ± 0.9	7.8 ± 0.5	5.4 ± 1.2	7.9 ± 0.4	7.6 ± 0.7
	Empathy (0 10)	1.3 ± 0.4	8.3 ± 1.1	8.5 ± 0.8	7.7 ± 0.9	7.9 ± 1.4	2.1 ± 0.9	0.8 ± 0.2
	Fear (0 10)	0.6 ± 0.1	3.2 ± 0.9	2.9 ± 1.1	3.1 ± 0.6	1.2 ± 0.4	6.8 ± 1.2	4.1 ± 1.1
	Irritation (0 10)	1.0 ± 0.4	3.2 ± 0.6	2.9 ± 0.8	3.0 ± 0.7	2.3 ± 0.5	4.3 ± 1.1	4.0 ± 0.9

Continued on next page

		Bird singing	Coughing	Vomiting	Crying	Laughter	Barking	Scratching
Schizophrenia	Pleasantness (−5 5)	0.9 ± 1.2	−1.8 ± 1.1	−1.5 ± 1.4	−2.1 ± 1.1	1.1 ± 0.7	−0.8 ± 0.7	−0.5 ± 0.3
	Arousal (0 10)	7.1 ± 1.0	4.9 ± 1.3	4.5 ± 0.8	4.6 ± 0.9	5.1 ± 1.0	8.7 ± 0.7	4.6 ± 0.8
	Empathy (0 10)	3.9 ± 1.4	1.3 ± 1.2	1.7 ± 0.6	1.2 ± 0.6	1.0 ± 1.4	2.5 ± 0.7	1.9 ± 0.7
	Fear (0 10)	3.9 ± 0.9	3.1 ± 1.0	1.9 ± 0.8	4.9 ± 1.2	2.9 ± 0.7	6.2 ± 0.7	2.4 ± 1.0
	Irritation (0 10)	4.5 ± 0.8	2.0 ± 1.2	1.7 ± 0.5	2.5 ± 0.8	4.3 ± 0.5	4.7 ± 0.9	2.3 ± 0.7
mTBI	Pleasantness (−5 5)	4.7 ± 0.2	−4.8 ± 0.6	−4.9 ± 0.2	−4.1 ± 0.3	−0.3 ± 0.9	−4.7 ± 0.3	−4.5 ± 0.8
	Arousal (0 10)	3.1 ± 0.4	8.8 ± 1.1	9.0 ± 0.9	8.8 ± 0.7	8.4 ± 0.7	7.2 ± 0.4	7.6 ± 0.7
	Empathy (0 10)	1.2 ± 0.6	2.0 ± 1.1	2.5 ± 0.8	1.9 ± 0.9	1.8 ± 1.0	1.3 ± 0.7	0.8 ± 0.2
	Fear (0 10)	0.9 ± 0.2	4.9 ± 0.9	4.6 ± 1.0	3.9 ± 0.8	3.0 ± 0.7	5.0 ± 1.0	4.1 ± 1.1
	Irritation (0 10)	6.2 ± 0.7	7.5 ± 0.8	6.8 ± 0.8	7.9 ± 0.6	7.3 ± 0.7	7.4 ± 0.9	7.7 ± 0.8
Stroke	Pleasantness (−5 5)	1.6 ± 0.7	−3.9 ± 0.8	−3.8 ± 0.9	−3.0 ± 0.3	2.7 ± 0.3	−2.1 ± 0.6	−2.9 ± 1.1
	Arousal (0 10)	4.1 ± 1.2	5.7 ± 0.9	6.0 ± 1.2	4.9 ± 0.9	6.3 ± 1.0	4.0 ± 0.5	4.7 ± 0.9
	Empathy (0 10)	1.1 ± 0.6	3.9 ± 1.2	3.6 ± 0.9	3.0 ± 0.8	7.1 ± 0.7	1.1 ± 0.6	1.2 ± 0.6
	Fear (0 10)	3.3 ± 0.7	3.0 ± 0.8	3.2 ± 1.3	2.2 ± 0.6	1.1 ± 0.6	6.8 ± 1.2	3.3 ± 1.0
	Irritation (0 10)	5.0 ± 0.9	3.2 ±1.6	3.0 ± 0.9	3.2 ± 0.9	2.9 ± 0.7	6.1 ± 1.1	3.4 ± 1.2

Note: Mean ± std.dev.

AIMS Press

© 2018 the Author(s), licensee AIMS Press. This is an open access article distributed under the terms of the Creative Commons Attribution License (http://creativecommons.org/licenses/by/4.0)
